# Evaluation of the Pfs25-IMX313/Matrix-M malaria transmission-blocking candidate vaccine in endemic settings

**DOI:** 10.1186/s12936-022-04173-y

**Published:** 2022-06-02

**Authors:** Charles Mulamba, Chris Williams, Katharina Kreppel, Jean Bosco Ouedraogo, Ally I. Olotu

**Affiliations:** 1 Interventions & Clinical Trials Department, Ifakara Health Institute, P.O. Box 74, Bagamoyo, Tanzania; 2 Nelson Mandela African Institution of Science and Technology, Tengeru, P. O. Box 447, Arusha, Tanzania; 3 The Jenner Institute, University of Oxford, Roosevelt Drive, Headington, Oxford, OX3 7DQ UK; 4 Institute of Research in Health Sciences, Bobo-Dioulasso, Burkina Faso

**Keywords:** Malaria, Vectors, Transmission-blocking vaccines

## Abstract

Malaria control relies heavily on the use of anti-malarial drugs and insecticides against malaria parasites and mosquito vectors. Drug and insecticide resistance threatens the effectiveness of conventional malarial interventions; alternative control approaches are, therefore, needed. The development of malaria transmission-blocking vaccines that target the sexual stages in humans or mosquito vectors is among new approaches being pursued. Here, the immunological mechanisms underlying malaria transmission blocking, status of Pfs25-based vaccines are viewed, as well as approaches and capacity for first in-human evaluation of a transmission-blocking candidate vaccine Pfs25-IMX313/Matrix-M administered to semi-immune healthy individuals in endemic settings. It is concluded that institutions in low and middle income settings should be supported to conduct first-in human vaccine trials in order to stimulate innovative research and reduce the overdependence on developed countries for research and local interventions against many diseases of public health importance.

## Background

The global malaria burden remains high; with an estimated 229 million cases and 409,000 deaths reported annually [1]. Over 90% of the malaria cases recorded in 2019 were in the sub-Saharan Africa, and Tanzania reported 5% of the global malaria deaths [2]. Malaria is caused by protozoan parasites of the genus *Plasmodium*, including *Plasmodium falciparum, Plasmodium vivax*, *Plasmodium ovale*, *Plasmodium knowlesi* and *Plasmodium malariae* [3]. The deadliest malaria parasite species, *P. falciparum*, is responsible for more than 97% of malaria mortality across sub-Saharan Africa. The available malaria control tools rely heavily on the use of anti-malarial drugs and insecticides against malaria parasites and mosquito vectors, respectively. However, standard malaria drugs do not kill malaria stages infectious to mosquitoes and the resistance of mosquito vectors to insecticides is a genuine threat to the current control strategies [4–7], particularly in areas where transmission is still high. While the current control methods have led to substantial reductions in malaria morbidity and mortality, progress has slowed [1].

Vaccination against malaria will ultimately be required for a sustainable impact on the disease burden and elimination. There has been progress towards vaccine development against the pre-erythrocytic and asexual malaria stages in humans in the last decade. However, variability and polymorphisms in target parasite proteins for the asexual stages present serious obstacles to breaking the parasites life cycle through vaccination [8]. Malaria elimination will, therefore, require new strategies that can thwart transmission. Malaria transmission-blocking vaccines offer a new approach targeting malaria parasites in the mosquito host [9], and would contribute significantly to malaria control and malaria elimination.

The *Plasmodium* parasite has a complex life cycle involving different stages between humans and female *Anopheles* mosquitoes. The parasite’s intracellular and extracellular survival is facilitated by a set of over 5000 parasite genes and specialized proteins which help it to grow and evade the hosts immune responses [10, 11]**.** The malaria parasite is well adapted to develop in different forms, including infectious forms to the human liver (sporozoites/pre-erythrocytic) and red blood cells (merozoite), as well as sexual/sporogonic stages (gametocyte/gametes/oocysts) in humans and mosquitoes. While clinical malaria results from parasite replication in human erythrocytes, it is the gametocytes, which are solely responsible for the spread of the disease. Each parasite development stage has a unique structure, morphology, surface proteins and metabolic pathways that keep changing as it progresses through its life cycle. These unique features also help the parasite to escape the host immune systems, creating challenges for drug and vaccine development [10]**.**

The WHO Global Vaccine Research Forum has set out a strategic framework for malaria vaccine development in The Malaria Vaccine Technology Roadmap, defining the goals for global malaria vaccine development community [12, 13]. The roadmap calls for the development of vaccines against the deadliest *Plasmodium* species by 2030 to achieve two key objectives: protective efficacy of at least 75 percent against clinical malaria and reduction of parasite transmission to substantially reduce the incidence of malaria infection, enabling elimination in multiple settings. Malaria vaccines in the pipeline are designed to target specific asexual or sporogonic/sexual stages of the malaria parasites in humans or mosquitoes. Liver stage vaccines like RTS-S and R21 are designed to elicit protection and prevent liver stage parasites to develop into blood stage parasites; thought to be done through both humoral as well as T-cell immune responses [14, 15]. Blood stage candidate vaccines such as reticulocyte homolog five (RH5) induce protection which reduces parasitaemia and, therefore, severity or episodes of the disease [16]. Malaria candidate vaccines developed to target the sexual stages of malaria parasites are commonly known as transmission-blocking vaccines (TBVs). These are designed to induce antibodies which neutralize parasites in the mosquito midgut after an infectious blood meal, consequently blocking onward transmission [17]. TBVs do not directly protect immunized individuals from clinical malaria but when deployed at community level can reduce the number of circulating infectious mosquitoes below a threshold that sustain transmission.

This review will discuss the foundational concepts and methodological approaches for evaluating Pfs25-IMX313 transmission-blocking candidate vaccine administered with Matrix M adjuvant in an endemic setting. The discussions will focus on *P. falciparum* since it forms the basis for designing the Pfs25-IMX313 vaccine candidate and is responsible for the majority of malaria mortality.

Vaccine development starts with identification and characterization of target antigens through scientific research. Potential vaccine candidates are then evaluated pre-clinically in animal models followed by clinical trial phases I–III in humans, to determine the safety, immunogenicity, and efficacy of the candidate vaccine, followed by regulatory approval and field deployment. Vaccine candidates are usually identified in laboratories with extensive infrastructure and cutting-edge technologies for conducting discovery research. Malaria vaccine research initially focused on the parasite stages leading to human infection (sporozoites/ pre-erythrocytic stages) and clinical disease (asexual stages), but as TBV development gained pace, the biological understanding of sexual stages has improved dramatically [18].

## Human-to-mosquito malaria transmission

Transmission of *P. falciparum* from humans to mosquitoes depends on the sexual phase of the parasite’s life cycle. The sexual cycle in humans starts with the activation of asexual schizonts to express the Apatella2-g gene (AP2-G), and production of sexual progeny, which become gametocytes [19, 20]. The AP2-G is member of the apicomplexan AP2 (APiAP2) family of DNA binding proteins, and highly conserved. *Plasmodium falciparum* gametocytes undergo five morphologically distinct forms (stages I-V) over a period of 10–12 days. The gametocyte stages I-IV sequester primarily in the bone marrow and spleen [21], before finding their way into blood circulation to complete the final maturation steps [22]. Stage V gametocytes can circulate for several weeks after clearance of asexual parasites [23] and must be taken up by a female *Anopheles* mosquito for the parasite sexual cycle to complete. Many factors influence the likelihood of gametocytes being transmitted to mosquitoes and establishing a successful mosquito stage infection [24]. General parasite characteristics that have been associated with differences in transmission potential and infectivity include gametocyte density [25–27], concurrent asexual parasite density [28], male to female gametocyte ratio [25, 29], and duration of infection [28, 30]**.** Host factors, including, age, anaemia, immunity, and mosquito factors are also known to influence gametocyte infectiousness [9].

In the mosquito midgut, *Plasmodium* gametocytes egress from the host erythrocyte and develop into gametes. Gametogenesis is induced by conditions in the mosquito midgut including; reduction in temperature, increase in pH and exposure to xanthurenic acid [31, 32]. Male gametocytes exflagellate producing up to eight motile microgametes; whereas, female gametocytes “round-up” to form one immotile macrogamete [33, 34]. Fertilization of a macrogamete by a microgamete result in the formation of a zygote, which then develops into a mature motile ookinete that traverses the midgut wall and forms an oocyst. Approximately 10–12 days after blood meal ingestion the rupture of oocysts results in the release of sporozoites, which will invade the mosquito salivary glands completing the mosquito stage of the *Plasmodium* life cycle [35].

## Parasite proteins targeted for blocking malaria transmission

Malaria transmission-blocking antigens are generally classified into two groups, pre-fertilization and the post-fertilization antigens. Pre-fertilization antigens are expressed during gametocyte development in humans, and contribute to the viability of mosquito stage parasites as well as playing a crucial role in fertilization [36, 37]. Some pre-fertilization antigens are gametocyte-specific while others are shared by the two sexes. Gametocyte-specific proteins remodel the human host cell to support gametocyte morphological development and maturation [38]**.** Pre-fertilization antigens shared by two gametes are involved in processes necessary for parasite colonization of the mosquito midgut. P48/45 and P230 are the most studied pre-fertilization antigens, belonging to the 6-cysteine protein family [39]. The “P” refers to *Plasmodium* and the number is the respective molecular weight of the protein on SDS-PAGE [40, 41]. On the other hand, P25 and P28 are the most advanced post-fertilization antigens [42, 43], expressed solely in the mosquito vector; though transcription may occur in circulating gametocytes [44]. The environment in mosquito midgut triggers the expression of post-fertilization antigens [45]. Pre-fertilization and post-fertilization proteins have homologs in all malaria species, and have been the focus of gametocyte research for decades as well as forming the basis of malaria transmission blocking vaccine (TBV) development [46, 47].

Some mosquito midgut parasite receptors previously reviewed [42, 43] have been proposed as variants of transmission blocking parasite antigens. It has been postulated that these mosquito-encoded receptors for specific parasite components can induce antibodies with potential to prevent mosquito infection and could also be targets for transmission blocking. The mosquito Pfs47-binding receptor [48] and the *Anopheles* alanyl aminopeptidase N (AnAPN1) are some of the examples of mosquito midgut proteins touted as targets for malaria transmission-blocking [49]. Other recently discovered antigens include a gametocyte plasma membrane protein Pbg37 and an ookinete surface protein PSOP25 that are believed to play a role on exflagelllation [50] and fertilization process [51, 52].

## *Plasmodium falciparum* surface antigen 25 (Pfs25)

Pfs25 is a cysteine-rich 217-amino acid composed of four tandem epidermal growth factor (EGF)-like domains and encoded by a 0.65-kb gene. Pfs25 is predicted to be a 25-kDa glycosylphosphatidylinositol (GPI)-anchored protein belonging to a 13-member P25 family of proteins [53]. The protein is involved in ookinete formation, survival in the mosquito midgut, and a possible role in parasite traversal of the mid-gut epithelium [54–56]. Based on structural analyses of the *P. vivax* ortholog Pvs25, the Pfs25 molecule is thought to be triangular and flat, and extensively expressed on the ookinete surface, forming a protective interlocking sheet [57, 58]. Pfs25 expression starts from the point when gametes egress from ingested red blood cells in the mosquito midgut, through the zygote and ookinete stages, with evidence of continued expression in the oocyst. The female gametocyte contains abundant Pfs25 transcripts, but its translation is repressed until transmission to the mosquito vector and egress from the gametocyte infected red blood cell [45]. Lack of expression in the human host means that Pfs25 has not come under the same level of immune pressure as many other potential malaria vaccine antigens [59]. Large-scale deep sequencing of *P. falciparum* field isolates from diverse geographic regions indicates that the pfs25 gene is highly conserved, revealing only one synonymous mutation [3]. It is hypothesized that Pfs25 vaccine–induced antibody responses should have transmission reducing activity against diverse parasitic strains. A study by Da et al. demonstrated that antibodies against recombinant Pfs25 had significant transmission reducing activity against diverse isolates from Burkina Faso and Thailand [60].

## Transmission-blocking immune mechanisms

Immune responses to pathogens generally involve humoral and cellular mechanisms**.** Humoral responses to parasite pre-fertilization antigens can be naturally acquired in the human host [61–67], while humoral responses against post-fertilization antigens expressed solely in the mosquito do not occur naturally in humans but can be induced by vaccination. Natural transmission-blocking antibodies occur because most of the gametocytes die in the human host, releasing intracellular proteins into the host circulation [68]. The released proteins are processed and presented to the human immune system resulting in induction of antibodies, which can cause a reduction or interruption of parasite development (fertilization) in the mosquitoes when taken up in a blood-meal containing gametocytes [69]. Antibodies against surface antigens on the gamete surface may prevent fertilization by opsonization resulting in immune cell-mediated lysis [70] or agglutination of gametes [71], or by direct lysis of gametes and activation of the complement system [72, 73]. Antibody activity against malaria parasite development in mosquitoes was first observed in 1958 in studies conducted using the avian *Plasmodium gallinaceum* and *Plasmodium fallax* in chickens and turkeys, with results showing that birds immunized with killed parasites had a significant and rapid fall in infectivity of gametocytes, supporting the role of active immunity [74]. These experiments were reproduced in 1976, and demonstrated that antibodies were indeed responsible for preventing parasite development in the mosquitoes midgut [75].

Naturally acquired antibody responses to *P. falciparum* gametocytes were first reported in individuals from malaria endemic sites in Papua New Guinea, where up to a 95% reduction in mosquito infectivity was observed during mosquito feeding experiments with cultured gametocytes [76]. The presence of naturally occurring antibodies in endemic areas has since been observed in studies from different countries including Tanzania [61, 62], Gambia, Kenya, Cameroon [63, 66, 77], Sri Lanka [64] and Burkina Faso [78]. Published data sets strongly suggest that recent gametocyte exposure is associated with a strong and effective transmission-blocking immunity (TBI) [66, 77, 79]. TBI has been significantly associated with antibody responses to pre-fertilization antigens: Pfs230, Pfs48/45, PfsHAP2 and to other novel gametocyte proteins [80, 81]. There have been conflicting observations regarding age as a factor of natural seroprevalence even in similar settings; in some studies, higher responses have been observed in older individuals [81] while this has not been the case in other studies even in similar study areas [67, 78].

The cellular immune mechanisms involved in the clearance of circulating gametocytes are not well understood. Since the red blood cells lack major histocompatibility complex molecules, direct targeting of gametocyte-infected erythrocytes by T lymphocytes is not possible. However, CD4 + T cells can respond to gametocyte antigens [82, 83] and appear capable of inducing long-lasting gametocytocidal immunity in rodent models [84].

Malaria parasite neutralization inside the mosquito is not only enabled by human-derived molecules but also by mosquito cellular immune components already reviewed [68]. Mosquito infection requires ingestion of both a male and a female gametocyte, whose microgametes and macrogametes must meet inside the midgut for fertilization. Studies have extensively characterized the population bottlenecks facing the malaria parasites in the mosquito-midgut indicating that only a small proportion of gametocytes ingested in the blood meal by the mosquito develop into oocysts; and about 38% of mosquitoes that take gametocytaemic blood become infected [9, 85, 86]. The parasite bottleneck is largely attributed to formation of a parasite physical barrier (peritrophic membrane or matrix), surrounding the blood meal after ingestion [87, 88]. The membrane prevents direct contact between the parasite and the midgut epithelium thereby interrupting the mosquito-midgut invasion [88]; although ookinetes are able to bleach this barrier through chitinase enzymes [89]. In addition, there are peroxidase and nitric oxide synthase present in epithelium, which leads to nitration of the gut epithelium with subsequent tagging of ookinetes for destruction by the complement system [90, 91].

## Status of Pfs25-based vaccines

Pfs25 is the most advanced transmission-blocking target protein [92] in the clinical pipeline, with Pfs230 the only other protein to be clinically evaluated. Because Pfs25 is not expressed in the vertebrate host, it is not subjected to natural immune selection pressure hence reduced risk of development of resistance parasite strains. However, Pfs25 cannot benefit from the natural boosting following vaccination.

The first clinical evaluation of the Pfs25 antigen was done in a Phase I/II trial that tested a multi-stage vaccine containing attenuated recombinant vaccinia viral vector encoding the sporozoite targets CSP and PfSSP2, the liver-stage target LSA1, and blood-stage antigens MSP1, SERA and AMA1 alongside Pfs25. The trial assessed the high and low doses of the vaccine and found the vaccine to be well tolerated. The Pfs25 component of the vaccine was highly immunogenic but the anti-Pfs25 antibodies did not show any transmission blocking activity [93].

In another study, the recombinant Pfs25 and the *P. vivax* homolog Pvs25, formulated with Montanide ISA 51 adjuvant (a water-in-oil emulsion) were tested in a single blinded, dose escalation-controlled Phase Ia trial. There was observed systemic reactogenicity, which was associated with the antigen/adjuvant combination. There had been no reports of previous severe systemic reactions for Montanide ISA 51 [94]. Nevertheless, 5/5 volunteers who had completed the vaccination before cessation of the trial developed substantial antibody responses to Pfs25, and the antibodies blocked parasite development in *Anopheles stephensi* mosquitoes. Transmission reduction activity (TRA) correlated with antibody titres, with serum from the best responder showing greater than 90% reduction in oocyst intensity [95].

Pfs25 has also been evaluated as protein-in-adjuvant formulation Pf25-EPA in Alhydrogel in a phase Ia trial [96]. Pfs25-EPA is a recombinant Pfs25 expressed in *Pichia pastoris*, chemically conjugated to detoxified ExoProtein A from *Pseudomonas aeruginosa*. The Pfs25-EPA/Alhydrogel vaccine was evaluated in a two-dose, three-dose, four-dose regimen, administered at zero, two, four and ten months, in thirty US healthy volunteers. The vaccine demonstrated a favourable safety profile in thirty healthy adult volunteers. At the highest dose, specific IgG responses were seen following the second and third vaccination, peaking two weeks after the 4th booster. In the two-dose regime, seroconversion rates were generally low with exception of one volunteer demonstrated high Pfs25 specific antibody titres after receiving a third booster dose. Antibody avidity was also shown to increase over successive vaccinations. In standardized membrane feeding assay (SMFA), significant TRA (greater than 50%) was demonstrated in the highest dose group after the fourth vaccination. Significant TRA was not detected in the majority of sera from time points following the second or third vaccination [96]. A further study on the safety and immunogenicity of the Pfs25-EPA/Alhydrogel vaccine, conducted in Bamako, Mali, was completed in 2018 and results of this Phase Ib trial are currently awaited (ClinicalTrials.gov Identifier: NCT01867463).

A Phase I dose-escalation study in the USA recently evaluated the safety and immunogenicity of virus-like particle (VLP) candidate Pfs25 VLP-FhCMB. Pfs25 VLP-FhCMB consist of Pfs25 genetically fused to the Alfalfa mosaic virus coat protein, and is produced in *Nicotiana benthamiana* plants [97]. Safety and immunogenicity of Pfs25 VLP-FhCMB, formulated in Alhydrogel, was evaluated in a three-dose regimen, administered on days 0, 56 and 168, in 44 healthy volunteers. The vaccine was well tolerated at all doses and specific IgG responses were induced one month after the second and third vaccinations. The two lower doses were generally not immunogenic. Statistically significant TRA was detected in the highest dose group, inhibiting oocyst intensity at close to 80% in a subset of 2/8 individuals receiving the 100 μg dose; however, overall, TRA was weak. Pfs25-based candidates in clinical developments are summarized in Table 1 [98].

**Table 1:** Status of the Pfs25-based candidate vaccines currently in clinical pipeline including the vaccine type and trial identification number

Vaccine candidate	Type	Stage of development	Trial Identification number/reference
Pfs25M-EPA/AS01	Subunit vaccine	Phase 1	NCT02942277
Pfs25EPA/Alhdrogel	Subunit vaccine	Phase 1	NCT02334462
Pfs25EPA/Alhdrogel	Subunit vaccine	Phase 1	NCT01867463
Pfs25 VLP-FhCMB	VLP vaccine	Phase 1	NCT02013687
ChAd63 Pfs25-IMX313 ± MVA Pfs25-IMX313	Viral vector & nanoparticle vaccine	Phase 1	NCT02532049
Pfs25 /Montanide ISA 51	Subunit vaccine	Phase 1	[95]
Pfs25-Pfs25	Conjugate vaccine	Phase 1	NCT00977899

The early Pfs25-based vaccine clinical trials have yielded modest and short-lived antibody responses with poor transmission-blocking activity, some have shown significant reactogenicity attributed to adjuvant formulations [42–44]. Several strategies are being pursued to overcome this hurdle, including advances in vaccine expression systems, delivery platforms, and adjuvant formulations. Expression in a variety of recombinant systems, including yeast [58, 99], plants [100], and algae [101], have been successful. Monoclonal antibodies raised against correctly folded recombinant Pfs25 antigens, such as the highly effective 4B7, have been found to achieve potent transmission blocking activity [102] at low concentrations [56, 99], and MAb 4B7 is used as a reference and positive control for mosquito-feeding assays [49, 103, 104]. Several approaches to vaccine particle development have also been pursued to increase immunogenicity. Conjugation to carriers is one of the advanced methods to improve vaccine immunogenicity where carrier proteins such as *Pseudomonas aeruginosa* exoprotein A (EPA) [105] and bacterial outer membrane protein complex (OMPC) have been used. Another method is fusion to partners that form complexes, generating particles [such as C4 bp oligomerization domain (IMX313)] expressed in *Escherichia coli* or modified lichenase carrier (LiKM) produced in *Nicotiana benthamiana*. Viral vector vaccines, such as Chad63/Modified Vaccinia Ankara, are also being assessed to improve immunogenicity [103, 106, 107] [107]. Adjuvants, such as Alhydrogel® and Montanide®, have been used for clinical trials of TBVs with some reactogenicity issues observed, and recently, the saponin-based Matrix-M adjuvant used for formulating the pre-erythrocytic vaccine R21[50], this would simplify future efforts to combine products [98].

## Pfs25-IMX313 vaccine development

Pfs25-IMX313 is a protein-nanoparticle vaccine for which the Pfs25 antigen is genetically fused to the IMX313 oligomerization domain [107]. The Pfs25 protein is based on the sequence from the 3D7 *P. falciparum* strain, with three potential N-linked glycosylation sites (112, 165 and 187) mutated. The recombinant protein-nanoparticle is expressed and secreted in the *Pichia pastoris* expression system [103].

IMX313 is a hybrid of the oligomerization domain of chicken complement inhibitor C4-binding protein (C4bp), with 21% homology to the sequence of the human protein (11 identical residues in an overlap of 52 amino acids) [107]. It is thus unlikely that vaccination with an antigen fused to IMX313 would generate an immune response against hC4bp. IMX313 forms homogenous, self-assembling heptamers of the antigen fused to it [107]. This C4bp oligomerization domain has been shown to spontaneously form soluble heptameric structures (termed nanoparticle in this study) when expressed in *E. coli*, and protein antigens fused to these domains induce higher antibody responses compared to the same amount of monomeric antigen. In addition, mice immunized with the blood-stage malaria vaccine candidate antigen MSP119 fused to IMX313 (expressed in *E. coli*) were protected against challenge with a lethal dose of *Plasmodium yoelii* parasites [107]. Immunization of mice and non-human primates with the *Mycobacterium tuberculosis* antigen 85A fused to IMX313 in both DNA vaccines and viral vectors also demonstrated that IMX313 enhances T-cells response, with a consistent increase in both CD4 + and CD8 + T cell responses.

Clinically, IMX313 was first assessed in the recombinant MVA85A-IMX313 tuberculosis vaccine candidate, administered intradermally, in a Phase Ia trial at the Jenner Institute in Oxford. In this first-in-human dose escalation study a total of 18 volunteers received either MVA85A-IMX313 or MVA85A. There were no safety concerns and no significant difference in the number of adverse events (AEs) between the MVA85A-IMX313 group and the MVA85A group. While there were no significant differences in immunogenicity between MVA85A and MVA85A-IMX313 groups [108]. Pfs25-IMX313 has been clinically tested using ChAd63 prime and MVA boost. Both vaccines had a favourable safety profile and induced both antibody and T-cell responses, but no significant TRA was observed.

## Pre-clinical evaluation of Pfs25-IMX313

Pfs25-IMX313 protein-in-adjuvant vaccines are immunogenic in BALB/c mice: Pfs25-IMX313, formulated in Alhydrogel, induced higher Pfs25-specifc IgG responses than soluble Pfs25 alone. These vaccine-induced antibodies were demonstrated to recognize native parasite proteins by immunofluorescence microscopy [103]. Vaccination with Pfs25-IMX313/Alhydrogel has been shown to induce antibody responses in BALB/c mice with significantly higher TRA compared to IgG from mice immunized with monomeric Pfs25/Alhydrogel (p < 0.02 at all concentrations of IgG tested). A similar effect was observed following vaccination with ChAd63/MVA Pfs25-IMX313, compared to viral vectors encoding Pfs25. Based on functional activity as assessed in SMFA, the quality of the anti-Pfs25 antibody response induced by immunization with the Pfs25-IMX313 protein-nanoparticle was significantly improved in comparison to that induced by soluble Pfs25 [103].

## Pfs25-IMX313 in Matrix-M

The clinical development of Pfs25-IMX313 in Matrix-M adjuvant is aimed towards the production of an effective transmission-blocking malaria vaccine for individuals in malaria-endemic regions. Matrix-M is a potent saponin-based adjuvant, comprising partially purified extracts of the bark of the *Quillaja saponaria* Molina tree, phosphatidylcholine and cholesterol, formulated as a 40 nm-sized complex. Matrix-M has been shown to efficiently activate and recruit immune cells to the draining lymph node, including T-cells, B-cells, NK-cells, dendritic cells and granulocytes, which may lead to enhanced antigen presentation [109]. Matrix-M has demonstrated a favourable safety profile, as well as the enhancement of cellular and humoral immune responses to a range of vaccines [102, 109, 110]. Available safety data demonstrates that the Matrix-M adjuvant is well tolerated and there have been no serious unexpected adverse reactions or adverse reactions of special interest reported [50, 102, 109, 111].

A Pfs25-IMX313 batch produced at GMP standard, and administered both with and without the Matrix-M adjuvant has been demonstrated to be immunogenic in BALB/c mice, and the addition of Matrix-M adjuvant led to significantly higher antibody responses [103]. Prime-boost in a two-dose schedule of Pfs25-IMX313 in Matrix-M adjuvant (P-P) was compared with heterologous viral-vectored prime-boost, with ChAd63 and MVA Pfs25-IMX313 (A-M), as well as with a mixed regimen, with ChAd63 Pfs25-IMX313 priming followed by Pfs25-IMX313/Matrix-M boost (A-P). The highest Pfs25-specific IgG titres were seen in PP prime-boost, which were significantly higher when compared with the ChAd63/MVA Pfs25-IMX313 regimen. In addition, antibody responses induced following immunization with Pfs25-IMX313 had higher avidity than responses induced by soluble Pfs25 [103].

## A brief discussion of Pfs25-IMX313/Matrix-M evaluation in Bagamoyo, Tanzania

One of the key steps in qualifying the viability of a candidate vaccine involves safety and tolerability evaluation in humans. An open label first-in-human phase I vaccination trial has been set up in Tanzania to assess the safety and tolerability of Pfs25-IMX313/Matrix-M in 52 semi-immune healthy volunteers from Bagamoyo district. Although each component of the Pfs25-IMX313/Matrix-M has been extensively tested in humans before, this will be the first time for all the vaccine components to be administered as “a single syringe”. Enrolment and vaccination shall start in adults before proceeding to children. Enrolment of volunteers will follow a strict staggered approach, with one of group of adults receiving a low dose of the vaccine followed by another adults’ group receiving a high dose of the vaccine in six-weeks interval. Vaccination in children is planned after completion of all the doses in adults and associated data review.

A two-years enrolment schedule has been designed, with one group of volunteers receiving immunization at months; zero, one and three, while another group shall receive immunization at months; zero, one and seven. This trial has been designed to only detect very large differences in the incidence of local and generalized adverse events between the vaccination groups. This is done to balance the chance to detect any possible untoward reactions against the desire to limit the number of volunteers involved for safety purposes, hence a small sample size per group will be used, given that similar studies evaluating Pfs25 candidate vaccine used between six and twenty participants per group [112, 113].

Bagamoyo is one of the six districts of the Tanzania’s coast region, with a population of about 345,000. It has a humid tropical climate with seasonal average temperature ranging from 13 ℃ to 30 ℃ and relative humidity as high as 98%. Malaria remains a public health problem in the district; and transmission tends to be highest between March–May and October–November [61]. Malaria transmission-blocking vaccines will mainly benefit endemic populations by reducing the malaria transmission in the vaccinated communities. While having limited benefit for tourists or military personnel since they do not offer direct benefit to the vaccinated individual. Their utility depends on mass deployment in the community and their abilities to protect whole populations against malaria transmission. Traditionally, assessment for safety and tolerability of new vaccines starts in naïve individuals in non-endemic settings during first-in-human phase one studies [98]. However, it is adventurous to conduct first in-human evaluations of Pfs25-IMX313/Matrix-M in endemic setting where transmission occurs is an ideal approach since the epidemiology, and the genetic make-up of the population may have a bearing on vaccine usage and general outcomes. [114, 115]. It is in line with the Belmont report that all the phases of the trial are conducted in populations who will benefit the most.

### Assessing immunogenicity

Vaccines work by ‘teaching’ the body to recognize a foreign invader (a pathogen) by priming the immune system after introduction of either part or an inactivated or weakened form of a pathogen and allowing the body to develop an effective response without danger of disease. This priming of the immune system means that, should the pathogen be encountered naturally, the immune system is able to react more quickly and effectively than if it had not been primed [116]. Immunogenicity is a complex measure of how well a vaccine works, and measures the type of immune response that the vaccine generates and its magnitude over time [117]. The analysis of vaccine immunogenicity provides valuable information not only on how well a vaccine is working, but can support aspects such as the determination of dosage and immunization schedules [118].

The overall level of induced antibodies can be measured through techniques such as ELISA (enzyme-linked immunosorbent serum assay), and specific neutralizing antibodies can be screened for via neutralizing assays. Measurement of T-cell responses can be more complex than the measurement of antibody levels, but through assays such as enzyme-linked Immunospot assays it is possible to define which types of T-cells are present and at what level. The immunogenicity of Pfs25-IMX313 administered with Matrix-M will be determined to assess if this candidate vaccine can induce stronger and more lasting transmission-blocking responses in humans compared to candidates tested previously. Levels of vaccine-induced Pfs25 antibodies will be measured in the immunized serum collected from vaccinated volunteers using Enzyme-Linked ImmunoSorbent Assays (ELISAs); the protocol described previously [119] will be adapted and followed when performing the assays. Other assays, including; ex vivo ELISpot for interferon gamma, flow cytometry, as well as specific ELISAs for measuring total IgG, isotype and avidity, will be performed. Sera samples with peak Pfs25 antibody levels will be selected for assessment of the activity/functionality of vaccine induced Pfs25 antibodies on oocyst development in mosquitoes.

### Approach for evaluating vaccine efficacy in Bagamoyo

Efficacy is a measure of the vaccine’s ability to prevent a clinical outcome of interest that can vary from infection, disease or mortality [118]. The standard epidemiologic method for TBV vaccine efficacy evaluation is a cluster randomized trial, involving two sets of community clusters, one set receiving a test vaccine and another set receiving a placebo with a follow up to determine and compare the incidence of malaria infections in the two sets of clusters. For TBVs, standard efficacy evaluation involves a number of villages, some receiving TBV and others receiving a control vaccine, with the readout being malaria cases [120]. However, this approach is difficult, time-consuming, and costly. The antibodies induced in humans by TBVs impact on parasite development in mosquitoes making it possible to indirectly measure of transmission blocking activity via mosquito feeding experiments and subsequent assessments of parasite infections in mosquitoes. Studies have indicated that a mosquito feeding experiment is an accurate proxy of the natural infection cycle in *P. falciparum*. This approach which is sometimes referred to as an ex-vivo efficacy evaluation can provide a platform to screen potential vaccine candidates and inform field development of the best candidate [24, 121]. This approach is ideal for preliminary efficacy evaluations during early stage TBV trials, as community-based efficacy evaluations cannot precede vaccine safety and immunogenicity qualification.

Mosquito membrane feeding assays (MFAs) which allow artificial mosquito infections with malaria, have been developed [122, 123], making it possible to evaluate the ability of naturally elicited or vaccine-induced antimalarial antibodies to block mosquito infection. Mosquito feeding can be performed either by direct biting on individuals infected with malaria or by ex vivo exposure to infected blood delivered through a glass membrane. While the first method requires the availability of infected patients carrying mature gametocytes in blood circulation and the fulfilment of ethical restrictions, the second is based on the use of cultured gametocytes, commonly known as Standard Membrane Feeding Assays (SMFAs) or the use of gametocyte-infected blood directly obtained from patients, usually referred to as Direct Membrane Feeding Assays (DMFAs). Transmission blocking activity can be assessed based on different indicators, such as reduction in the number of infected mosquitoes, reduction in oocyst counts per mosquito, or by the reduction of sporozoite production [124].

**Fig. 1:**
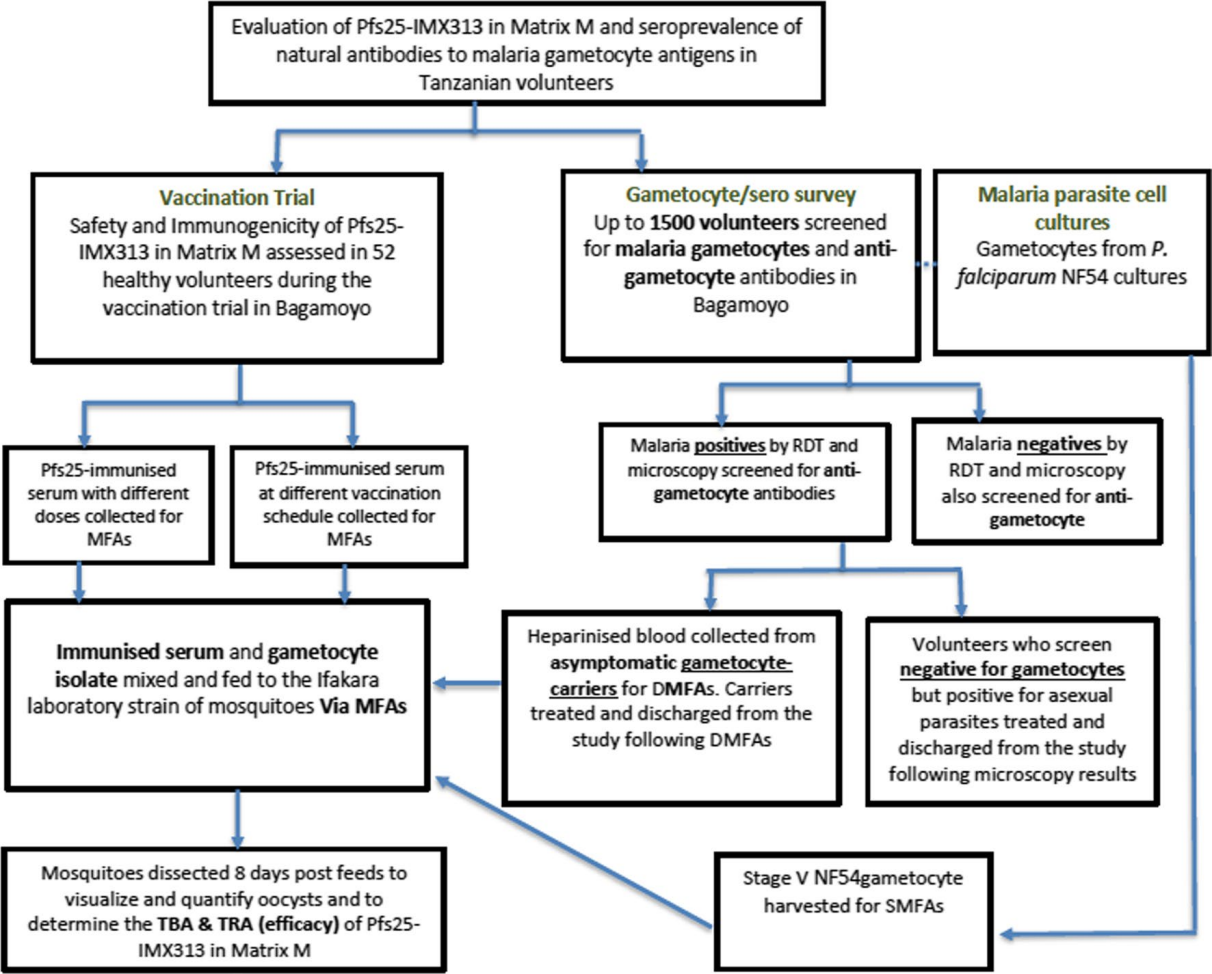
Layout for evaluation of Pfs25-IMX313in Matrix-M; Pfs25-immunized serum samples from vaccinated volunteers will be collected and mixed with field or cultured gametocyte isolates and fed to laboratory-reared mosquitoes to determine the ex-vivo efficacy of the vaccine candidate in form of transmission blocking [102] and reducing [50] activities

The ex-vivo efficacy of Pfs25-IMX313 candidate vaccine in Bagamoyo will be evaluated using SMFAs and DMFAs as summarized in Fig. 1. Immunized sera with peak antibody levels will be mixed with wild or cultured gametocyte isolates and fed to a laboratory-reared [125] mosquito colony, to determine whether the vaccine-induced Pfs25 antibodies can reduce or prevent oocyst development inside mosquito-midguts. SMFAs will be performed using gametocytes of NF54 laboratory strain of *P. falciparum* parasite [126]. During Direct membrane feeding assays, the autologous plasma of gametocyte carriers will be replaced with Pfs25-immune serum from vaccinated volunteers. Gametocyte carrier plasma will be replaced with *Plasmodium*-naive European/American serum in control assays. The protocol described previously [60] will be used with two main modifications; the gametocyte-infected venous blood will not be washed in order to maintain some autologous blood components, and Pfs25 immunized serum will be used instead of immunized plasma. The mosquitoes will be dissected eight days post-feeding to detect and quantify midgut infections with *P. falciparum* oocysts.

Two parameters, transmission-blocking and -reducing activities (TBA and TRA respectively) will be used to determine ex-vivo efficacy of the Pfs25-IMX313/Matrix M candidate vaccine with both field and laboratory gametocyte isolates. TBA is a measure of percent (%) inhibition of oocyst prevalence and will be determined as: 100 X {1 – (proportion of mosquitoes with any oocysts in the test assays)/ (proportion of mosquitoes with any oocysts in the control assays). TRA is measure of percent (%) inhibition of mean oocyst intensity and will be calculated as: 100 X {1 − (mean number of oocysts in the test assays)/(mean number of oocysts in the control assays)} [127]. If use of microscopy for detection of oocyst proves to be insufficient, infections will be diagnosed using molecular techniques.

## Evidence of natural transmission-blocking immunity in Bagamoyo

Understanding the dynamics of natural transmission-reducing immune responses will aid future local deployment of transmission-blocking vaccines [61, 62]. Therefore, in addition to evaluating activity of Pfs25 vaccine-induced responses, we will investigate the natural prevalent anti-gametocytes antibodies in Bagamoyo and the extent of transmission-reducing activity of prevalent antibodies. A sero-survey has been planned in villages around Bagamoyo districts starting August 2021. The survey will mainly target volunteers in the age group 5–14 years because these are known to harbour a greater number of gametocytes and more infectious [128], but other volunteers between the age of 15–45 will also be enrolled. Screening for natural antibodies will mainly be conducted using gametocyte and gamete antigens; Pfs230 and Pfs48/45, Pfs47. The sero-survey will also be used to identify gametocyte carriers who will donate field gametocytes to be challenged against Pfs25 vaccine-induced antibodies from the vaccination trial. Transmission-blocking activity will be assessed against cultured gametocytes and a laboratory strain of local mosquitoes.

## Capacity and infrastructural demands

### Facilities and resources

Good infrastructure and strong capacities are required to accomplish vaccine testing and evaluations activities. Vaccination of participants is a major undertaking involving; community engagement and sensitization, screening and vaccination of trial participants, close safety monitoring, laboratory investigations and biological assays, such as the measurement of immune responses and functionality via ELISAs and mosquito membrane-feeding assay (MFAs). This undertaking requires a team of multidisciplinary professionals with diverse expertise to match different aspects the work. Community liaison officers together with the village health care workers or trained field worker are required to engage communities and prospective study volunteers, clinicians are needed to assess volunteers for inclusion/exclusion criteria and monitor their health after receiving the vaccination, additionally pharmacists are required to oversee vaccine preparation, physicians and nurses who administer the vaccines must be present, and ACLS or PALS trained clinicians who provide monitoring and care for any post-immunization emergency are a must. Also needed are: medical biotechnologists to ensure proper sample collection, processing, transport, and storage as well as immediate measurement of biological parameters, as well as medical entomologists to perform mosquito-feeding assays and associated dissections for endpoint analysis, data managers to enter, and quality assurance managers and ensure participants’ safety and data quality according to established procedures [98].

The trial will be conducted at the Clinical Trial Facility (CTF) located at the IHI Branch in Bagomoyo- coastal region, Tanzania. The IHI has close partnerships with the Tanzania government, and a number of reputable regional and northern partners including Kenya Medical Research Institute-Wellcome Trust Research Programme (KWTRP), Institute of Research in Health Sciences (IRSS), Burkina Faso, Jenner Institute at the University of Oxford, The Swiss Tropical and Public Health Institute (Swiss TPH), and the US National Institutes of Health (NIH), among others. The CTF has clinical consultation rooms, a pharmacy, vaccination room, data management room and an observation area for study participants. There is a resuscitation room that is equipped with oxygen, suction, defibrillator and resuscitation kits. The trial facility has experienced trained staff including pharmacist, physicians and highly experienced nurses. Study physician and nurses are ACLS, and PALS certified and are involved in regular re-training activities involving simulations. A clinician is accessible 24 h a day by phone to provide medical care to participants as needed outside the scheduled study visits. The Bagamoyo District Hospital (BDH), a public hospital is in close proximity for secondary level health care services. The BDH has one paediatric and a general wards, basic radiology and ultrasound facility and routine paediatric surgery is also carried. Referral to tertiary level facilities is rarely required, but, in cases where such referrals are needed, participants are sent to Muhimbili National Hospital or Aga Khan Hospital in Dar es Salaam. The CTF is supported by Bagamoyo Research and Training Centre (BRTC) laboratory located within the grounds of BDH. Medical laboratory investigations and testing in the fields of; clinical chemistry, haematology, parasitology, biochemistry, microbiology, immunology analyses are performed in BRTC laboratory. The BRTC laboratory is ISO 15189:2012 accredited by the Southern African Development Community Accreditation Services (SADCAS). The CTF is also supported by well-equipped and resourced insectary with capacity to do high-throughput rearing of mosquitoes, mosquito-feeding assays and dissections. In addition, the institute has a good transportation network to ensure constant transportation of persons, samples, and materials between the field, clinics, and laboratories. There is also an independent Quality Assurance unit in place, which oversees the quality and integrity during trials.

As a testimony for its ability to conduct first-in-human studies, the CTF hosted the first [129] study in Africa in 2012 evaluating its safety and feasibility in malaria endemic population. The challenge studies require a very close safety monitoring to ensure the safety and wellbeing of study participants is assured.

## Ethics and community engagement

All required approvals have been granted for study activities to be conducted. The Tanzania National Guidelines of Ethics for Health Research, stipulate regulatory approval for clinical trials, starting with approvals from ethics committees at respective host Institutions where the studies will be undertaken. If the study involves testing an investigational drug or vaccine, institutional approval must be followed by clearance from National Medicines & Medical Devices Authority (TMDA) before final approval from the ethics committee at National Institute of Medical Research (NIMR). Local authorities and community representatives including village heads, family heads, health providers, and school heads are consulted for permission and approval conduct clinical trials in the communities. A series of meetings are held to explain the study to the potential participants in the community. These meetings include sensitization for general information and pre-screening for more detailed information. Locally acceptable approaches are used to inform and invite community representatives, potential participants, and parents/guardians to the sensitization meetings. A lay background and justification of the intended study is discussed in a culturally appropriate fashion. Attention is focused on study procedures and blood sample collections. The purpose of blood sample collection and the associated risks are explained in a culturally sensitive fashion, to avoid misunderstandings. Opportunities are provided for the community members to ask questions and get responses from the Investigators. At the end of a sensitization meeting, potential participants are invited to register and attend a pre-screening meeting at CTF, where detailed information about the study is provided using the PIS in a group and individual sessions.

This participative approach has reinforced community confidence towards our studies and subsequently positioned IHI to successfully conduct previous clinical trials [129–132] in Tanzania. The success of these trials was a result of IHI’s technical capacities and dynamic team, to execute the intense activities that compose these trials.

## Challenges and considerations

### Technical challenges

Mosquito feeding assays are cumbersome and problematic to standardize. In addition, human antibodies must act quickly before degradation in the mosquito and before parasite traversal of midgut epithelium (~ 24 h) that limits its accessibility to antibody [98] but it is difficult to determine the exact ingested antibody levels in the mosquito required for a subsequent transmission blockade. Transmission blocking and reducing activities directly measured in the human host will overcome these challenges and strengthen the current efforts to develop transmission-blocking vaccines [133, 134]. The need for the Pfs25-based vaccines to maintain high levels of potent antibody that preclude mosquito infection and lack of natural boosting within the human host continues to be major concern for the vaccine development [98].

### Reagents and supplies

There are limited local suppliers for many essentials, reagents and consumables required for field, clinical and laboratory activities during field trials. Consequently, the local supply chain largely depends on overseas suppliers and northern partners. The limitation on movements brought about by the COVID-19 pandemic and the prioritization for production and delivery of COVID-19 related medical supplies have hugely disrupted local supply chains for interventions of other disease, including; malaria, HIV and TB, among other infections. The current disruptions in local and international supply chains and the complex local procurement policies have caused significant delays in the implementation of the trials in Tanzania. To address this, collaborations with overseas institutions have been strengthened and new suppliers of a range of consumables found. Furthermore, the IHI is working to address some of the internal challenges that are contributing to the delayed procurement of reagents by restructuring the supply chain unit and improving the processes and human resource capacity.

## Conclusion

Transmission blocking vaccines can play a significant role in reducing the burden of malaria and their field evaluation requires experienced personnel and infrastructure to provide credible results. There has been remarkable improvement and strengthening of research capacity and infrastructure across many southern institutions in recent decades. These institutions should be given more opportunities to conduct first-in human trials to stimulate innovative research and reduce the overdependence on developed countries for research and local interventions against many diseases of public health importance. Malaria transmission occurs largely in low- and middle-income countries, where the disease epidemiology, and the genetics of the endemic population will have significant impact on use of the vaccine and the desired outcomes. Relying on results from first-in human evaluations conducted in naïve settings may not yield relevant information on the safety and immunogenicity for a vaccine that will eventually be deployed in endemic settings.

## Data Availability

No applicable.

## References

[1] WHO. World malaria report (2020). 20 years of global progress and challenges.

[2] WHO. World malaria report 2019. Geneva, World Health Organization,2019.

[3] Manske M, Miotto O, Campino S, Auburn S, Almagro-Garcia J, Maslen G (2012). Analysis of *Plasmodium falciparum* diversity in natural infections by deep sequencing. Nature.

[4] Muller P, Warr E, Stevenson BJ, Pignatelli PM, Morgan JC, Steven A (2008). Field-caught permethrin-resistant *Anopheles gambiae* overexpress CYP6P3, a P450 that metabolises pyrethroids. PLoS Genet.

[5] Ramphul U, Boase T, Bass C, Okedi LM, Donnelly MJ, Muller P (2009). Insecticide resistance and its association with target-site mutations in natural populations of *Anopheles gambiae* from eastern Uganda. Trans R Soc Trop Med Hyg.

[6] Wondji CS, Irving H, Morgan J, Lobo NF, Collins FH, Hunt RH (2009). Two duplicated P450 genes are associated with pyrethroid resistance in *Anopheles funestus*, a major malaria vector. Genome Res.

[7] Hemingway J, Ranson H (2000). Insecticide resistance in insect vectors of human disease. Annu Rev Entomol.

[8] Riley EM, Stewart VA (2013). Immune mechanisms in malaria: new insights in vaccine development. Nat Med.

[9] Smith RC, Vega-Rodríguez J, Jacobs-Lorena M (2014). The *Plasmodium* bottleneck: malaria parasite losses in the mosquito vector. Mem Inst Oswaldo Cruz.

[10] Florens L, Washburn MP, Raine JD, Anthony RM, Grainger M, Haynes JD (2002). A proteomic view of the *Plasmodium falciparum* life cycle. Nature.

[11] Greenwood BM, Fidock DA, Kyle DE, Kappe SH, Alonso PL, Collins FH (2008). Malaria: progress, perils, and prospects for eradication. J Clin Invest.

[12] Moorthy VS, Newman RD, Okwo-Bele JM (2013). Malaria Vaccine Technology Roadmap. Lancet.

[13] WHO. Malaria Vaccine Technology Roadmap. Geneva, World Health Organization, 2006.

[14] Vanderberg JP, Nussenzweig RS, Most H, Orton CG. Protective immunity produced by the injection of x-irradiated sporozoites of *Plasmodium berghei*. II. Effects of radiation on sporozoites. J Parasitol. 1968:1175–1180. 5757691

[15] Reyes-Sandoval A, Wyllie DH, Bauza K, Milicic A, Forbes EK, Rollier CS (2011). CD8+ T effector memory cells protect against liver-stage malaria. J Immunol.

[16] Fried M, Duffy PE (2015). Designing a VAR2CSA-based vaccine to prevent placental malaria. Vaccine.

[17] Coutinho-Abreu IV, Ramalho-Ortigao M (2010). Transmission blocking vaccines to control insect-borne diseases: a review. Mem Inst Oswaldo Cruz.

[18] de Jong RM, Tebeje SK, Meerstein-Kessel L, Tadesse FG, Jore MM, Stone W (2020). Immunity against sexual stage *Plasmodium falciparum* and *Plasmodium vivax* parasites. Immunol Rev.

[19] Sinha A, Hughes KR, Modrzynska KK, Otto TD, Pfander C, Dickens NJ (2014). A cascade of DNA-binding proteins for sexual commitment and development in *Plasmodium*. Nature.

[20] Kafsack BF, Rovira-Graells N, Clark TG, Bancells C, Crowley VM, Campino SG (2014). A transcriptional switch underlies commitment to sexual development in malaria parasites. Nature.

[21] Joice R, Nilsson SK, Montgomery J, Dankwa S, Egan E, Morahan B, et al. *Plasmodium falciparum* transmission stages accumulate in the human bone marrow. Sci Transl Med. 2014;6:244re245. 10.1126/scitranslmed.3008882 PMC4175394 25009232

[22] Aguilar R, Magallon-Tejada A, Achtman AH, Moraleda C, Joice R, Cisteró P (2014). Molecular evidence for the localization of *Plasmodium falciparum* immature gametocytes in bone marrow. Blood.

[23] Reuling IJ, Van De Schans LA, Coffeng LE, Lanke K, Meerstein-Kessel L, Graumans W (2018). A randomized feasibility trial comparing four antimalarial drug regimens to induce *Plasmodium falciparum* gametocytemia in the controlled human malaria infection model. Elife.

[24] Bousema T, Drakeley C (2011). Epidemiology and infectivity of *Plasmodium falciparum* and *Plasmodium vivax* gametocytes in relation to malaria control and elimination. Clin Microbiol Rev.

[25] Bradley J, Stone W, Da DF, Morlais I, Dicko A, Cohuet A (2018). Predicting the likelihood and intensity of mosquito infection from sex specific *Plasmodium falciparum* gametocyte density. Elife.

[26] Da DF, Churcher TS, Yerbanga RS, Yaméogo B, Sangaré I, Ouedraogo JB (2015). Experimental study of the relationship between *Plasmodium* gametocyte density and infection success in mosquitoes; implications for the evaluation of malaria transmission-reducing interventions. Exp Parasitol.

[27] Collins KA, Wang CY, Adams M, Mitchell H, Rampton M, Elliott S (2018). A controlled human malaria infection model enabling evaluation of transmission-blocking interventions. J Clin Invest.

[28] Ouédraogo LA, Gonçalves BP, Gnémé A, Wenger EA, Guelbeogo MW, Ouédraogo A (2016). Dynamics of the human infectious reservoir for malaria determined by mosquito feeding assays and ultrasensitive malaria diagnosis in Burkina Faso. J Infect Dis.

[29] Paul RE, Brey PT, Robert V (2002). *Plasmodium* sex determination and transmission to mosquitoes. Trends Parasitol.

[30] Johnston GL, Smith DL, Fidock DA (2013). Malaria's missing number: calculating the human component of R0 by a within-host mechanistic model of *Plasmodium falciparum* infection and transmission. PLoS Comput Biol.

[31] Billker O, Lindo V, Panico M, Etienne A, Paxton T, Dell A (1998). Identification of xanthurenic acid as the putative inducer of malaria development in the mosquito. Nature.

[32] Billker O, Miller A, Sinden R (2000). Determination of mosquito bloodmeal pH in situ by ion-selective microelectrode measurement: implications for the regulation of malarial gametogenesis. Parasitology.

[33] Sinden R (1983). Sexual development of malarial parasites. Adv Parasitol.

[34] Sinden R (1983). The cell biology of sexual development in *Plasmodium*. Parasitology.

[35] Meis J, Wismans P, Jap P, Lensen A, Ponnudurai T (1992). A scanning electron microscopic study of the sporogonic development of *Plasmodium falciparum* in *Anopheles stephensi*. Acta Trop.

[36] van Dijk MR, Janse CJ, Thompson J, Waters AP, Braks JA, Dodemont HJ (2001). A central role for P48/45 in malaria parasite male gamete fertility. Cell.

[37] Eksi S, Czesny B, Van Gemert GJ, Sauerwein RW, Eling W, Williamson KC (2006). Malaria transmission-blocking antigen, Pfs230, mediates human red blood cell binding to exflagellating male parasites and oocyst production. Mol Microbiol.

[38] Tibúrcio M, Niang M, Deplaine G, Perrot S, Bischoff E, Ndour PA (2012). A switch in infected erythrocyte deformability at the maturation and blood circulation of *Plasmodium falciparum* transmission stages. Blood.

[39] Gerloff DL, Creasey A, Maslau S, Carter R (2005). Structural models for the protein family characterized by gamete surface protein Pfs230 of *Plasmodium falciparum*. Proc Natl Acad Sci USA.

[40] Kaushal D, Carter R, Rener J, Grotendorst C, Miller L, Howard R (1983). Monoclonal antibodies against surface determinants on gametes of *Plasmodium gallinaceum* block transmission of malaria parasites to mosquitoes. J Immunol.

[41] Rener J, Graves PM, Carter R, Williams JL, Burkot TR (1983). Target antigens of transmission-blocking immunity on gametes of *Plasmodium falciparum*. J Exp Med.

[42] Nikolaeva D, Draper SJ, Biswas S (2015). Toward the development of effective transmission-blocking vaccines for malaria. Expert Rev Vaccines.

[43] Wu Y, Sinden RE, Churcher TS, Tsuboi T, Yusibov V (2015). Development of malaria transmission-blocking vaccines: from concept to product. Adv Parasitol.

[44] Miao J, Fan Q, Parker D, Li X, Li J, Cui L (2013). Puf mediates translation repression of transmission-blocking vaccine candidates in malaria parasites. PLoS Pathog.

[45] Mair GR, Braks JA, Garver LS, Wiegant JC, Hall N, Dirks RW (2006). Regulation of sexual development of *Plasmodium* by translational repression. Science.

[46] Carter R, Miller L, Rener J, Kaushal D, Kumar N, Graves PM (1984). Target antigens in malaria transmission blockling immunity. Philos Trans R Soc Lond B Biol Sci.

[47] Vermeulen AN, Ponnudurai T, Beckers P, Verhave J, Smits M, Meuwissen J (1985). Sequential expression of antigens on sexual stages of *Plasmodium falciparum* accessible to transmission-blocking antibodies in the mosquito. J Exp Med.

[48] Molina-Cruz A, Canepa GE, Kamath N, Pavlovic NV, Mu J, Ramphul UN (2015). *Plasmodium* evasion of mosquito immunity and global malaria transmission: The lock-and-key theory. Proc Natl Acad Sci USA.

[49] Miura K, Takashima E, Deng B, Tullo G, Diouf A, Moretz SE (2013). Functional comparison of *Plasmodium falciparum* transmission-blocking vaccine candidates by the standard membrane-feeding assay. Infect Immun.

[50] Datoo MS, Natama MH, Somé A, Traoré O, Rouamba T, Bellamy D (2021). Efficacy of a low-dose candidate malaria vaccine, R21 in adjuvant Matrix-M, with seasonal administration to children in Burkina Faso: a randomised controlled trial. Lancet.

[51] Liu F, Li L, Zheng W, He Y, Wang Y, Zhu X (2018). Characterization of *Plasmodium berghei* Pbg37 as both a pre- and postfertilization antigen with transmission-blocking potential. Infect Immun.

[52] Wang J, Zheng W, Liu F, Wang Y, He Y, Zheng L (2017). Characterization of Pb51 in *Plasmodium berghei* as a malaria vaccine candidate targeting both asexual erythrocytic proliferation and transmission. Malar J.

[53] Kaslow DC, Quakyi IA, Syin C, Raum MG, Keister DB, Coligan JE (1988). A vaccine candidate from the sexual stage of human malaria that contains EGF-like domains. Nature.

[54] Baton LA, Ranford-Cartwright LC (2005). Do malaria ookinete surface proteins P25 and P28 mediate parasite entry into mosquito midgut epithelial cells?. Malar J.

[55] Tomas AM, Margos G, Dimopoulos G, van Lin LH, de Koning-Ward TF, Sinha R (2001). P25 and P28 proteins of the malaria ookinete surface have multiple and partially redundant functions. EMBO J.

[56] Miura K, Keister DB, Muratova OV, Sattabongkot J, Long CA, Saul A (2007). Transmission-blocking activity induced by malaria vaccine candidates Pfs25/Pvs25 is a direct and predictable function of antibody titer. Malar J.

[57] Saxena AK, Singh K, Su HP, Klein MM, Stowers AW, Saul AJ (2006). The essential mosquito-stage P25 and P28 proteins from *Plasmodium* form tile-like triangular prisms. Nat Struct Mol Biol.

[58] Stowers AW, Keister DB, Muratova O, Kaslow DC (2000). A region of *Plasmodium falciparum* antigen Pfs25 that is the target of highly potent transmission-blocking antibodies. Infect Immun.

[59] Kaslow DC, Quakyi IA, Keister DB (1989). Minimal variation in a vaccine candidate from the sexual stage of *Plasmodium falciparum*. Mol Biochem Parasitol.

[60] Da DF, Dixit S, Sattabonkot J, Mu J, Abate L, Ramineni B (2013). Anti-Pfs25 human plasma reduces transmission of *Plasmodium falciparum* isolates that have diverse genetic backgrounds. Infect Immun.

[61] Bousema T, Roeffen W, Meijerink H, Mwerinde H, Mwakalinga S, van Gemert G-J (2010). The dynamics of naturally acquired immune responses to *Plasmodium falciparum* sexual stage antigens Pfs230 & Pfs48/45 in a low endemic area in Tanzania. PLoS ONE.

[62] Bousema J, Drakeley C, Kihonda J, Hendriks J, Akim N, Roeffen W (2007). A longitudinal study of immune responses to *Plasmodium falciparum* sexual stage antigens in Tanzanian adults. Parasite Immunol.

[63] Mulder B, Lensen T, Tchuinkam T, Roeffen W, Verhave JP, Boudin C (1999). *Plasmodium falciparum*: membrane feeding assays and competition ELISAs for the measurement of transmission reduction in sera from Cameroon. Exp Parasitol.

[64] Premawansa S, Gamage-Mendis A, Perera L, Begarnie S, Mendis K, Carter R (1994). *Plasmodium falciparum* malaria transmission-blocking immunity under conditions of low endemicity as in Sri Lanka. Parasite Immunol.

[65] Roeffen W, Lensen T, Mulder B, Teelen K, Sauerwein R, Eling W (1994). Transmission blocking immunity as observed in a feeder system and serological reactivity to Pfs 48/45 and Pfs230 in field sera. Mem Inst Oswaldo Cruz.

[66] Drakeley C, Eling W, Teelen K, Bousema J, Sauerwein R, Greenwood B (2004). Parasite infectivity and immunity to *Plasmodium falciparum* gametocytes in Gambian children. Parasite Immunol.

[67] Drakeley C, Bousema J, Akim N, Teelen K, Roeffen W, Lensen A (2006). Transmission-reducing immunity is inversely related to age in *Plasmodium falciparum* gametocyte carriers. Parasite Immunol.

[68] Kengne-Ouafo JA, Sutherland CJ, Binka FN, Awandare GA, Urban BC, Dinko B (2019). Immune responses to the sexual stages of *Plasmodium falciparum* parasites. Front Immunol.

[69] Mendis K, David P, Carter R (1990). Human immune responses against sexual stages of malaria parasites: considerations for malaria vaccines. Int J Parasitol.

[70] Ranawaka G, Alejo-Blanco A, Sinden R (1994). Characterization of the effector mechanisms of a transmission-blocking antibody upon differentiation of *Plasmodium berghei* gametocytes into ookinetes in vitro. Parasitology.

[71] Tachibana M, Ishino T, Tsuboi T, Torii M (2018). The *Plasmodium yoelii* microgamete surface antigen (PyMiGS) induces anti-malarial transmission blocking immunity that reduces microgamete motility/release from activated male gametocytes. Vaccine.

[72] Grotendorst C, Carter R, Rosenberg R, Koontz L. Complement effects on the infectivity of *Plasmodium gallinaceum* to *Aedes aegypti* mosquitoes. I. Resistance of zygotes to the alternative pathway of complement. J Immunol. 1986;136:4270–4. 3517168

[73] Healer J, McGuinness D, Hopcroft P, Haley S, Carter R, Riley E (1997). Complement-mediated lysis of *Plasmodium falciparum* gametes by malaria-immune human sera is associated with antibodies to the gamete surface antigen Pfs230. Infect Immun.

[74] Huff CG, Marchbank DF, Shiroishi T. Changes in infectiousness of malarial gametocytes. II. Analysis of the possible causative factors. Exp Parasitol. 1958;7:399–417. 10.1016/0014-4894(58)90036-5 13562104

[75] Carter R, Chen DH (1976). Malaria transmission blocked by immunisation with gametes of the malaria parasite. Nature.

[76] Graves PM, Carters R, Burkot TR, Quakyi IA, Kumar N (1988). Antibodies to *Plasmodium falciparum* gamete surface antigens in Papua New Guinea sera. Parasite Immunol.

[77] Bousema T, Sutherland CJ, Churcher TS, Mulder B, Gouagna LC, Riley EM (2011). Human immune responses that reduce the transmission of *Plasmodium falciparum* in African populations. Int J Parasitol.

[78] Ouédraogo AL, Roeffen W, Luty AJ, de Vlas SJ, Nebie I, Ilboudo-Sanogo E (2011). Naturally acquired immune responses to *Plasmodium falciparum* sexual stage antigens Pfs48/45 and Pfs230 in an area of seasonal transmission. Infect Immun.

[79] Ouédraogo AL, Eckhoff PA, Luty AJ, Roeffen W, Sauerwein RW, Bousema T (2018). Modeling the impact of *Plasmodium falciparum* sexual stage immunity on the composition and dynamics of the human infectious reservoir for malaria in natural settings. PLoS Pathog.

[80] Jones S, Grignard L, Nebie I, Chilongola J, Dodoo D, Sauerwein R (2015). Naturally acquired antibody responses to recombinant Pfs230 and Pfs48/45 transmission blocking vaccine candidates. J Infection.

[81] Stone WJ, Campo JJ, Ouédraogo AL, Meerstein-Kessel L, Morlais I, Da D (2018). Unravelling the immune signature of *Plasmodium falciparum* transmission-reducing immunity. Nat Commun.

[82] Riley EM, Ong C, Olerup O, Eida S, Allen S, Bennett S, et al. Cellular and humoral immune responses to *Plasmodium falciparum* gametocyte antigens in malaria-immune individuals. Limited response to the 48/45-kilodalton surface antigen does not appear to be due to MHC restriction. J Immunol. 1990;144:4810–6. 2112574

[83] Goodier MR, Targett G (1997). Evidence for CD4+ T cell responses common to *Plasmodium falciparum* and recall antigens. Int Immunol.

[84] Harte P, Rogers N, Targett G (1985). Role of T cells in preventing transmission of rodent malaria. Immunology.

[85] Gouagna LC, Mulder B, Noubissi E, Tchuinkam T, Verhave JP, Boudin C (1998). The early sporogonic cycle of *Plasmodium falciparum* in laboratory-infected *Anopheles gambiae*: an estimation of parasite efficacy. Trop Med Int Health.

[86] Sinden RE, Billingsley PF (2001). *Plasmodium* invasion of mosquito cells: hawk or dove?. Trends Parasitol.

[87] Dinglasan R, Devenport M, Florens L, Johnson J, McHugh C, Donnelly-Doman M (2009). The *Anopheles gambiae* adult midgut peritrophic matrix proteome. Insect Biochem Mol Biol.

[88] Shao L, Devenport M, Jacobs-Lorena M (2001). The peritrophic matrix of hematophagous insects. Arch Insect Biochem Physiol.

[89] Vinetz JM, Dave SK, Specht CA, Brameld KA, Xu B, Hayward R (1999). The chitinase PfCHT1 from the human malaria parasite *Plasmodium falciparum* lacks proenzyme and chitin-binding domains and displays unique substrate preferences. Proc Natl Acad Sci USA.

[90] Kumar S, Gupta L, Han YS, Barillas-Mury C (2004). Inducible peroxidases mediate nitration of anopheles midgut cells undergoing apoptosis in response to *Plasmodium* invasion. J Biol Chem.

[91] Garver LS, de Almeida OG, Barillas-Mury C (2013). The JNK pathway is a key mediator of *Anopheles gambiae* antiplasmodial immunity. PLoS Pathog.

[92] Coelho CH, Doritchamou JYA, Zaidi I, Duffy PE (2017). Advances in malaria vaccine development: report from the 2017 malaria vaccine symposium. NPG Vaccines.

[93] Ockenhouse CF, Sun PF, Lanar DE, Wellde BT, Hall BT, Kester K (1998). Phase I/IIa safety, immunogenicity, and efficacy trial of NYVAC-Pf7, a pox-vectored, multiantigen, multistage vaccine candidate for *Plasmodium falciparum* malaria. J Infect Dis.

[94] Malkin EM, Durbin AP, Diemert DJ, Sattabongkot J, Wu Y, Miura K (2005). Phase 1 vaccine trial of Pvs25H: a transmission blocking vaccine for *Plasmodium vivax* malaria. Vaccine.

[95] Wu Y, Ellis RD, Shaffer D, Fontes E, Malkin EM, Mahanty S (2008). Phase 1 trial of malaria transmission blocking vaccine candidates Pfs25 and Pvs25 formulated with montanide ISA 51. PLoS ONE.

[96] Talaat KR, Ellis RD, Hurd J, Hentrich A, Gabriel E, Hynes NA (2016). Safety and immunogenicity of Pfs25-EPA/Alhydrogel(R), a transmission blocking vaccine against *Plasmodium falciparum*: an open label study in malaria naive adults. PLoS ONE.

[97] Chichester JA, Green BJ, Jones RM, Shoji Y, Miura K, Long CA (2018). Safety and immunogenicity of a plant-produced Pfs25 virus-like particle as a transmission blocking vaccine against malaria: a Phase 1 dose-escalation study in healthy adults. Vaccine.

[98] Doumbo OK, Niaré K, Healy SA, Sagara I, Duffy PE. Malaria transmission-blocking vaccines: present status and future perspectives. In: Towards malaria elimination-a leap forward. Manguin S, Dev V (Eds). IntechOpen. 2018.

[99] Barr PJ, Green KM, Gibson HL, Bathurst IC, Quakyi IA, Kaslow DC (1991). Recombinant Pfs25 protein of *Plasmodium falciparum* elicits malaria transmission-blocking immunity in experimental animals. J Exp Med.

[100] Farrance CE, Chichester JA, Musiychuk K, Shamloul M, Rhee A, Manceva SD (2011). Antibodies to plant-produced *Plasmodium falciparum* sexual stage protein Pfs25 exhibit transmission blocking activity. Hum Vaccin.

[101] Patra KP, Li F, Carter D, Gregory JA, Baga S, Reed SG (2015). Alga-produced malaria transmission-blocking vaccine candidate Pfs25 formulated with a human use-compatible potent adjuvant induces high-affinity antibodies that block *Plasmodium falciparum* infection of mosquitoes. Infect Immun.

[102] Cox RJ, Pedersen G, Madhun AS, Svindland S, Sævik M, Breakwell L (2011). Evaluation of a virosomal H5N1 vaccine formulated with Matrix M™ adjuvant in a phase I clinical trial. Vaccine.

[103] Li Y, Leneghan DB, Miura K, Nikolaeva D, Brian IJ, Dicks MD (2016). Enhancing immunogenicity and transmission-blocking activity of malaria vaccines by fusing Pfs25 to IMX313 multimerization technology. Sci Rep.

[104] Miura K, Stone WJ, Koolen KM, Deng B, Zhou L, van Gemert G-J (2016). An inter-laboratory comparison of standard membrane-feeding assays for evaluation of malaria transmission-blocking vaccines. Malar J.

[105] Qian F, Wu Y, Muratova O, Zhou H, Dobrescu G, Duggan P (2007). Conjugating recombinant proteins to *Pseudomonas aeruginosa* ExoProtein A: a strategy for enhancing immunogenicity of malaria vaccine candidates. Vaccine.

[106] Wu Y, Przysiecki C, Flanagan E, Bello-Irizarry SN, Ionescu R, Muratova O (2006). Sustained high-titer antibody responses induced by conjugating a malarial vaccine candidate to outer-membrane protein complex. Proc Natl Acad Sci USA.

[107] Ogun SA, Dumon-Seignovert L, Marchand J-B, Holder AA, Hill F (2008). The oligomerization domain of C4-binding protein (C4bp) acts as an adjuvant, and the fusion protein comprised of the 19-kilodalton merozoite surface protein 1 fused with the murine C4bp domain protects mice against malaria. Infect Immun.

[108] Minhinnick A, Satti I, Harris S, Wilkie M, Sheehan S, Stockdale L (2016). A first-in-human phase 1 trial to evaluate the safety and immunogenicity of the candidate tuberculosis vaccine MVA85A-IMX313, administered to BCG-vaccinated adults. Vaccine.

[109] Bengtsson KL, Song H, Stertman L, Liu Y, Flyer DC, Massare MJ (2016). Matrix-M adjuvant enhances antibody, cellular and protective immune responses of a Zaire Ebola/Makona virus glycoprotein (GP) nanoparticle vaccine in mice. Vaccine.

[110] Venkatraman N, Anagnostou N, Bliss C, Bowyer G, Wright D, Lövgren-Bengtsson K (2017). Safety and immunogenicity of heterologous prime-boost immunization with viral-vectored malaria vaccines adjuvanted with Matrix-M™. Vaccine.

[111] Lövgren Bengtsson K, Morein B, Osterhaus AD (2011). ISCOM technology-based Matrix M™ adjuvant: success in future vaccines relies on formulation. Expert Rev Vaccines.

[112] Afolabi MO, Tiono AB, Adetifa UJ, Yaro JB, Drammeh A, Nébié I (2016). Safety and immunogenicity of ChAd63 and MVA ME-TRAP in West African children and infants. Mol Ther.

[113] Sagara I, Healy SA, Assadou MH, Gabriel EE, Kone M, Sissoko K (2018). Safety and immunogenicity of Pfs25H-EPA/Alhydrogel, a transmission-blocking vaccine against *Plasmodium falciparum*: a randomised, double-blind, comparator-controlled, dose-escalation study in healthy Malian adults. Lancet Infect Dis.

[114] Haralambieva IH, Salk HM, Lambert ND, Ovsyannikova IG, Kennedy RB, Warner ND (2014). Associations between race, sex and immune response variations to rubella vaccination in two independent cohorts. Vaccine.

[115] Nédélec Y, Sanz J, Baharian G, Szpiech ZA, Pacis A, Dumaine A (2016). Genetic ancestry and natural selection drive population differences in immune responses to pathogens. Cell.

[116] Kerr JR, Freeman AL, Marteau TM, van der Linden S (2021). Effect of information about COVID-19 vaccine effectiveness and side effects on behavioural intentions: two online experiments. Vaccines.

[117] Hodgson SH, Mansatta K, Mallett G, Harris V, Emary KR, Pollard AJ (2021). What defines an efficacious COVID-19 vaccine? A review of the challenges assessing the clinical efficacy of vaccines against SARS-CoV-2. Lancet Infect Dis.

[118] WHO. Guidelines on clinical evaluation of vaccines: regulatory expectations. WHO Technical Rep Ser. 2004;924.

[119] Miura K, Orcutt AC, Muratova OV, Miller LH, Saul A, Long CA (2008). Development and characterization of a standardized ELISA including a reference serum on each plate to detect antibodies induced by experimental malaria vaccines. Vaccine.

[120] Nunes JK, Woods C, Carter T, Raphael T, Morin MJ, Diallo D (2014). Development of a transmission-blocking malaria vaccine: progress, challenges, and the path forward. Vaccine.

[121] Bousema T, Dinglasan RR, Morlais I, Gouagna LC, van Warmerdam T, Awono-Ambene PH (2012). Mosquito feeding assays to determine the infectiousness of naturally infected *Plasmodium falciparum* gametocyte carriers. PLoS ONE.

[122] Ponnudurai T, Lensen AH, Van Gemert GJ, Bensink MP, Bolmer M, Meuwissen JH (1989). Infectivity of cultured *Plasmodium falciparum* gametocytes to mosquitoes. Parasitology.

[123] Arévalo-Herrera M, Solarte Y, Rocha L, Álvarez D, Beier JC, Herrera S (2011). Characterization of *Plasmodium vivax* transmission-blocking activity in low to moderate malaria transmission settings of the Colombian Pacific coast. Am J Trop Med Hyg.

[124] Vallejo AF, Rubiano K, Amado A, Krystosik AR, Herrera S, Arevalo-Herrera M (2016). Optimization of a membrane feeding assay for *Plasmodium vivax* infection in *Anopheles albimanus*. PLoS Negl Trop Dis.

[125] Benedict M. Methods in *Anopheles* research. MR4, Malaria Research and Reference Reagent Resource Center, Atlanta, USA. 2009.

[126] Miura K, Deng B, Tullo G, Diouf A, Moretz SE, Locke E (2013). Qualification of standard membrane-feeding assay with *Plasmodium falciparum* malaria and potential improvements for future assays. PLoS ONE.

[127] Miura K, Swihart BJ, Deng B, Zhou L, Pham TP, Diouf A (2016). Transmission-blocking activity is determined by transmission-reducing activity and number of control oocysts in *Plasmodium falciparum* standard membrane-feeding assay. Vaccine.

[128] Churcher TS, Bousema T, Walker M, Drakeley C, Schneider P, Ouédraogo AL (2013). Predicting mosquito infection from *Plasmodium falciparum* gametocyte density and estimating the reservoir of infection. Elife.

[129] Shekalaghe S, Rutaihwa M, Billingsley PF, Chemba M, Daubenberger CA, James ER (2014). Controlled human malaria infection of Tanzanians by intradermal injection of aseptic, purified, cryopreserved *Plasmodium falciparum* sporozoites. Am J Trop Med Hyg.

[130] Jongo SA, Shekalaghe SA, Church LP, Ruben AJ, Schindler T, Zenklusen I (2018). Safety, immunogenicity, and protective efficacy against controlled human malaria infection of *Plasmodium falciparum* sporozoite vaccine in Tanzanian adults. Am J Trop Med Hyg.

[131] Jongo SA, Church LP, Mtoro AT, Schindler T, Chakravarty S, Ruben AJ (2020). Increase of dose associated with decrease in protection against controlled human malaria infection by PfSPZ Vaccine in Tanzanian adults. Clin Infect Dis.

[132] Metta E, Mahumane SF, Sitali DC, Nyamhanga T, Mboera LE, Frumence G, et al. Response of the social systems to Covid-19 in Mozambique, Tanzania and Zambia: a synthesis of the challenges and opportunities.

[133] Gebru T, Ajua A, Theisen M, Esen M, Ngoa UA, Issifou S (2017). Recognition of *Plasmodium falciparum* mature gametocyte-infected erythrocytes by antibodies of semi-immune adults and malaria-exposed children from Gabon. Malar J.

[134] Tonwong N, Sattabongkot J, Tsuboi T, Iriko H, Takeo S, Sirichaisinthop J (2012). Natural infection of *Plasmodium falciparum* induces inhibitory antibodies against gametocyte development in human hosts. Jpn J Infect Dis.

